# Taiwan Green Propolis Nanoparticles Induce Antiproliferation and Apoptosis in Oral Cancer Cells

**DOI:** 10.3390/biomedicines13040921

**Published:** 2025-04-09

**Authors:** Wen-Da Huang, Shu-Fen Peng, Nai-Wen Tsao, Sheng-Yang Wang, Shu-Ling Tzeng, Nien-Jen Hu

**Affiliations:** 1 Institute of Biochemistry, College of Life Sciences, National Chung Hsing University, Taichung 402202, Taiwan; 2 Department of Medical Research, China Medical University Hospital, Taichung 404327, Taiwan; 3 Program in Specialty Crops and Metabolomics, Academy of Circle Economy, National Chung Hsing University, Nantou City 540225, Taiwan; 4 Department of Forestry, National Chung Hsing University, Taichung 402202, Taiwan; 5 Institute of Medicine, Chung Shan Medical University, Taichung 40203, Taiwan; 6 Department of Medical Research, Chung Shan Medical University Hospital, Taichung 402306, Taiwan; 7 PhD Program in Translational Medicine, National Chung Hsing University, Taichung 402202, Taiwan

**Keywords:** TGP, propolis, prenylflavonoid, oral cancer, nanoparticle, zein, *Macaranga tanarius*, apoptosis

## Abstract

**Introduction:** Taiwan green propolis (TGP) is rich in prenylflavonoids and exhibits antioxidant, antibacterial, antiviral, and antitumour properties. It induces apoptosis in various cancer cells, making it a highly promising natural medicine. Although the health benefits and food applications of TGP are widely recognised, no study has explored its effects on Taiwan oral cancer cells (OECM1). This study investigated whether TGP induces apoptosis in OECM1 cells. **Methods:** High-performance liquid chromatography (HPLC), thin-layer chromatography, and liquid chromatography/mass spectrometry were used to identify the components in TGP and the fruit peel of *Macaranga tanarius*. The inhibitory activities of TGP dissolved in DMSO (TGP_DMSO_) and encapsulated in food-grade zein nanoparticles (TGP_NP_) against OECM1 cells were compared using MTT assays. The morphological changes, cell cycle analysis, and protein expression profiles of OECM1 cells after the TGP treatments were performed using microscopy, flow cytometry, and Western blot, respectively. **Results:** An MTT assay of TGP_DMSO_-treated OECM1 cells suggested an IC_50_ of 12.6 µg/mL, demonstrating that TGP_DMSO_ exhibits significant cytotoxicity. Subsequent MTT assays revealed TGP_NP_’s cytotoxicity against OECM1 with an IC_50_ of 11.6 µg/mL. Flow cytometry revealed that TGP_NP_ induced a cell arrest in S phase and DNA fragmentation. Western blotting analyses manifested an increase in Bax and cl-Casp9 and a decrease in Bcl2 and PARP. **Conclusion:** This study demonstrated that both TGP_DMSO_ and TGP_NP_ treatments induced apoptosis in OECM1 cells with a comparable IC_50_. Notably, utilising edible zein as a nanoparticle carrier for TGP mitigates the cytotoxicity risk associated with DMSO, providing a novel and safe approach for cancer treatment.

## 1. Introduction

The approximately 20,000 species of Hymenoptera insects are the primary pollinators on Earth. Honeybees, bumblebees, and stingless bees in the Apoidea superfamily are all representative social insects [1]. They exhibit communal behaviours such as collecting nectar, secreting beeswax, and storing pollen [2]. Notably, propolis, another important bee product, is collected by approximately 600 species of stingless bees and *Apis mellifera* (honeybee) or hybrids of honeybees worldwide [3]. Propolis is a resinous substance that bees collect from plant exudates, tender buds, or fruit skins [4]. Bees carry this resinous material back to the hive using the pollen baskets on their hind legs. The primary functions of the resinous substance are to repair cracks and holes in the hive, reinforce structures, and prevent predator invasion [5].

The health benefits of propolis are attributed to the rich content of plant secondary metabolites, known as phytochemicals [6]. The compounds are biosynthesised through the mevalonic acid/polyketide pathways in plants and are categorised into seven major groups: flavonoids, coumarins, triphenols, diphenols, terpenoids, phytosterols, and phenylpropanoids. Previous research indicates that these compounds are the primary bioactive components of medicinal plants [7]. These compounds have also been shown to be present in propolis [8]. Propolis collected from different regions around the world exhibits a diverse range of pharmacological activities, including anti-*Staphylococcus aureus*, antifungal, antiviral, anti-inflammatory, cavity-preventive, immunostimulatory, antitumour, radioprotective, and free radical scavenging properties [9,10].

The composition of propolis depends greatly on the plant sources at the collection site, the geographical location, and the bee species involved. Currently, the most studied and renowned propolis is the Brazilian green propolis (BGP), whose main functional compounds are artepillin C and kaempferol [11]. Studies on propolis and related products from Europe have focused on flavonoids and total polyphenol content. In the past 20 years, research in East Asia, especially Taiwan and Japan, has mainly focused on local propolis. The propolis collected by honeybees in Okinawa, Japan, comes from the resin in the fruit peels of *Macaranga* [12,13]. This ingredient has excellent anti-free-radical properties and anticancer effects [14]. Research during the same period found that nine main compounds were identified in Taiwan green propolis (TGP), mainly containing isoprenoid flavonoids (namely, propolins) [15]. Although the origin is different, the molecular composition and structure are very similar. The extractable flavonoid content of TGP can exceed 65%; thus, TGP has the highest plant polyphenol content among all propolis products, according to the literature to date [15]. TGP is effective in combating liver cancer, lung adenocarcinoma, brain cancer, breast cancer, and colorectal cancer [16]. Hence, TGP is of great potential for the development of antitumour drugs. Additionally, TGP can be formulated into food for oral administration, offering potential effectiveness in oral cancer treatment. Therefore, this study chose oral squamous carcinoma cells (OECM1) as the target cancer cells [17].

The treatment of cancer can be divided into four stages: in stage I, cancer is potentially curable by surgical excision, while stages II to IV generally require treatments such as chemotherapy, radiotherapy, and immunotherapy [18,19]. The primary reason for chemotherapy failure is the inherent or acquired multidrug resistance (MDR) of cancer cells. The mechanism of MDR involves the efflux of drugs through the transport proteins, making it difficult for the drugs to destroy cancer cells effectively [20]. Previous findings indicate that several ABC transporters are involved in MDR, including P-glycoprotein (MDR1/ABCB1), multidrug resistance protein (MRP1/ABCC1), and breast cancer resistance protein (ABCG2/BCRP/MXR/ABCP) [21]. These transporters, which are ATPase enzymes, are located on the cell membrane and utilise energy from ATP hydrolysis to expel various drugs from the cell. It has been demonstrated that paclitaxel loaded in the synthetic polymer nanoparticles circumvents the drug efflux pathways in cancer cells [22]. Thus, selecting appropriate drug carriers to reduce MDR and enhance drug uptake has become one of the key aspects of cancer therapy.

Nanoparticle drug carriers are a highly popular technology in drug delivery. Cellular drug uptake preferences are influenced by factors such as shape, size, surface charge, and tumour microenvironment [23]. Among these factors, the size of nanoparticles is the most critical. Nanoparticles in the 120–150 nm size range are internalised via clathrin- or caveolin-mediated endocytosis [24]. Furthermore, nanoparticles within this size range permeate more effectively and accumulate in tumours due to the enhanced permeability and retention (EPR) effect [25], ensuring controlled and sustained drug release at the therapeutic level. This drug delivery strategy has proven effective in both neoadjuvant and adjuvant immunotherapies utilising immune checkpoint inhibitors [26]. Drug carriers can be made from metal particles, polymeric polymers, and lipid emulsions [27,28]. For practical applications, key considerations include ease of use, edibility, degradability, and high physiological compatibility, making zein-based biomaterials suitable for nanoparticle development. Due to its unique amino acid sequence and physicochemical properties, zein has self-assembly capabilities and can be transformed into films, fibres, and micro/nanostructures through solvent adjustment [29]. In the food and pharmaceutical industries, zein is currently utilised as a nanodelivery system; this drug carrier has been used to transport drugs and nutrients for enhancing their bioactivity. For example, it has been used to encapsulate lipophilic compounds such as curcumin, vitamin D, fish oil, DNA, enzymes, and polyphenols [30,31]. Furthermore, zein enhances stability against environmental changes in the gastrointestinal tract and increases bioavailability, and most importantly, zein is non-cytotoxic to intestinal cells, offering excellent safety and convenience for pharmaceutical applications [32]. In this study, we analysed the components of TGP using TLC. We compared the cytotoxicity of TGP in OECM1 cells using TGP prepared with dimethyl sulfoxide (DMSO) and zein nanoparticles and investigated the molecular mechanism of cytotoxicity. TGP prepared in zein nanoparticles not only inhibits the proliferation and induces the apoptosis of OECM1 cells but also offers significant advantages in terms of edibility and safety.

## 2. Materials and Methods

### 2.1. Acquisition and Extraction of TGP and Fruit Peel of Macaranga tanarius

In central Taiwan, from May to August 2021, 5 g of raw green propolis and *Macaranga tanarius* fruit peel resin (MTF, Yuanzhang, Taiwan, 23.39020° N, 120.18565° E) were collected for extraction with ethanol (EtOH) at a 1:9 ratio [33]. The mixture was added to a centrifuge tube and extracted on an orbital shaker at room temperature for 3 h, after which it was left to stand overnight. Subsequently, the centrifuge tube was shaken once more to disperse the contents, and the mixture was then centrifuged at 1500 rpm for 10 min to separate the resin components. Then, the supernatant was collected, and the extract was stored in a freezer at −20 °C overnight before undergoing lyophilisation. Prior to experimentation, the extract was redissolved in methanol or EtOH. For the cell cytotoxicity analysis, TGP was precisely weighed and dissolved in EtOH at a concentration of 100 mg/mL and then diluted ten-fold with 9 mL of DMSO to prepare a TGP solution (TGP_DMSO_) at 10 mg/mL.

### 2.2. Compound Analysis of Taiwan Green Propolis

Thin-layer chromatography (TLC) involves the use of the solvent concentration ratio and the affinity differences in molecules to estimate the solvent concentration ratio required for the separation of compounds. In this study, we observed the distribution of the main components of natural substances at an absorbance of 254 nm. The solvents used were methanol (Honeywell Co., Charlotte, NC, USA), n-hexane (Honeywell Co.), and ethyl acetate (Honeywell Co.). After drying and concentrating, the TGP and samples were weighed, and an appropriate amount was dissolved in ethyl acetate or methanol to obtain a 1% solution. A baseline was drawn on a thin silicone sheet, and samples were applied to the silicone sheet by using a capillary tube. The silicone sheet was then placed in chromatographic liquid (n-hexane/ethyl acetate/methanol, 6:3:1, code HEMe) for 15 min. The silicone sheet was removed, air-dried, and observed and photographed under UV light. HPLC (Simadzu, Tokyo, Japan) was conducted initially with a methanol 85% solution, which was increased to 90%; gradient elution was conducted for 30 min, with clear separation of the peaks of each component of TGP. Liquid chromatography/mass spectrometry (LC/MS, Bruker, Billerica, MA, USA) was then performed to analyse the molecular weight of each peak.

### 2.3. MTT Assay of Cell Viability

In this study, the OECM-1 cells were provided by Professor Muh-Hwa Yang from the National Yang-Ming Chiao Tung University. The cells were seeded in 10 cm culture dishes in RPMI-1640 (Gbico, Waltham, MA, USA) plus 10% foetal bovine serum (FBS, Cytiva, Westborough, MA, USA) and 100 unit/mL penicillin–streptomycin (Hyclone, Logan, UT, USA). The environment in the incubator was set at 37 °C and 5% CO_2_. Using a 24-well plate, 1 × 10^4^ cells were seeded into 500 μL of culture medium per well and cultured overnight. After thorough mixing, the solution was diluted to half of its concentration. Subsequently, 5 mL of the solution was transferred into another centrifuge tube for further dilution and mixing, and TGP_DMSO_ or nanoparticle culture media at concentrations of 2.5, 5, 10, 20, and 40 µL/mL were prepared. After the culture medium was removed from the 24-well plate, 500 µL TGP culture media of varying concentrations were added and incubated for 24 and 48 h. Subsequently, in the dark, 80 µL of the 3-[4,5-dimethylthiazol-2-yl]-2,5 diphenyl tetrazolium bromide (MTT, Sigma-Aldrich, St. Louis, MO, USA) reagent was added to each well of the 24-well plate; this was followed by further incubation for 30 min. After removing the culture medium, 200 µL of DMSO was added to each well and shaken for 5 min. The DMSO solution from the 24-well plate was then transferred into a 96-well plate, and absorbance was measured at 570 nm on an ELISA reader (TECAN sunrise, Männedorf, Switzerland). Cell viability was calculated based on the absorbance values.

### 2.4. Cell Cycle Assay Using Flow Cytometry

In this study, we inoculated 2 × 10^5^ cells in a 10 cm culture dish; this was followed by incubation at 37 °C. After the cells were stabilised, we removed the culture medium and replaced it with medium containing different concentrations of TGP. Following incubation for 48 h, we removed the culture medium and transferred the cells to a centrifuge tube. The cells were then rinsed once with 2 mL phosphate-buffered saline (PBS, Biomate, Taipei, Taiwan) and added to a centrifuge tube; 1 mL of trypsin was added, and the mixture was incubated in a cell culture incubator for 3 min. The mixture was centrifuged at 1500 rpm for 5 min, and the supernatant was removed. Following this, 2 mL of PBS was added to the centrifuge tube, which was gently tapped to disperse the cells and centrifuged for another 8 min. After the removal of the supernatant, the tube was placed on a shaker, 2 mL of ice-cold 75% alcohol was dripped in, and the sample was stored at −20 °C overnight. Afterwards, the tube was centrifuged for 5 min; the supernatant was removed and placed into an empty test tube, and 300–500 µL of propidium iodide (PI, Sigma, St. Louis, MO, USA) was added. The test tube was then wrapped in aluminium foil to protect it from light, and the reaction was allowed to proceed for 30 min. Finally, cell cycle analysis was performed. The mixture for PI staining consisted of 10 mL PBS (containing 0.1% Triton X-100) + 2 mg of DNAse-free RNAse (final concentration: 200 µg/mL) + 0.40 mL of PI Stock (final concentration: 20 µg/mL).

### 2.5. Apoptosis Marker Protein Extraction

In this study, we inoculated 2 × 10^5^ cells in a 10 cm culture dish; this was followed by incubation in a cell culture incubator for 24 h until the cells were fully adhered to the dish. We then removed the culture medium and added 10 mL of TGP_DMSO_ or TGP nanoparticle culture medium at doses of 0, 5, 10, and 15 µg/mL, with incubation for another 48 h. After thoroughly removing the culture medium and adding 1 mL of PBS to shake and disperse the cells, we removed the PBS. We then added 100 µL of radioimmunoprecipitation assay buffer (RIPA, Waltham, MA, USA) to the dish, ensuring its even distribution. A cell scraper was used to collect the cell fluid into a centrifuge tube, which was then placed in a refrigerator at −20 °C overnight. The next day, the cells were removed from the refrigerator and thawed before vortexing for 10 s; the cells were then centrifuged for 10 s and chilled for 5 min. The aforementioned steps were repeated three times. Afterwards, ultracentrifugation was conducted at 12,000 rpm for 30 min at 4 °C. The supernatant was collected for protein quantification.

### 2.6. Protein Quantification

We dispensed 2 µL of bovine serum albumin (BSA) at concentrations of 0, 0.125, 0.25, 0.5, 1.0, and 2.0 µg/mL into the wells of a 96-well plate. Then, we added 2 µL of the collected protein extract to the plate. To each well, 198 µL of protein assay dye (Bio-Rad, Singapore) was added, and the reaction was allowed to proceed for 5 min. Afterwards, a spectrophotometer was used to detect absorbance at 595 nm. Using the BSA standards, the concentration trend line and slope were derived and were used to estimate the protein concentration of the samples. After protein quantification, the protein concentration was adjusted to 25 µg/mL, and 5 µL of 5× dye and RIPA were added. After thorough mixing by shaking, the plate was incubated at 100 °C for 5 min and then stored at −20 °C.

### 2.7. Acrylamide Protein Electrophoresis and Transfer

We placed 12% acrylamide (Bio-Rad, Singapore) gel in an electrophoresis tank (Bio-Rad, Singapore), poured in glycine running buffer, and loaded 30 µL of the protein sample per well (70 V for 30 min and then increasing to 120 V for 60 min). Subsequently, we removed the gel, placed it on filter paper, and then covered it with a methanol-activated PVDF membrane. Sponges and filter paper were placed on top, and the gel was secured on a black clamp holder. The entire setup was then placed in an ice-cooled electrophoretic transfer tank (Bio-Rad, Singapore). We then poured the transfer buffer, installed the electrodes, and set the current to 300 mA for 60 min. After electrophoresis, we removed the gel and the membrane placed it in buffer (0.5 g of milk powder dissolved in 10 mL TBST buffer) on a shaker for 1 h. The membrane was then rinsed with TBST for 10 min three times. Next, we cut the membrane into slices and then placed them separately in 5 mL β-tubulin (1:500, iReal, Taipei, Taiwan) and apoptosis protein-related antibodies (1:500, iReal, Taipei, Taiwan) on a shaker overnight at 4 °C. After three rinses with TBST, HRP-conjugated secondary antibody (iReal, Taipei, Taiwan) was added on a shaker for 1 h at room temperature. Subsequently, two rinses with TBST were conducted. To photograph the protein bands, we prepared 1 mL of the electrochemiluminescence (ECL, Bio-Rad, Singapore) mixing reagent and used an LAS4000 chemiluminescence camera (Supplementary Figure S1).

### 2.8. Nanoprocessing of Taiwan Green Propolis Using Zein

The nanoparticle materials were prepared with a zein (ACROS, Waltham, MA, USA)/TGP/gamma-polyglutamic acid (γ-PGA, Vedan, Taichung, Taiwan) ratio of 5:1:1 (*w*/*w*/*w*). The stock solutions used were 50 mg/mL zein (Zein_EtOH_) and 3 mg/mL TGP in 80% ethanol (TGP_EtOH_), and 5 mg/mL γ-PGA in ddH_2_O (γ-PGA) at pH 7.0 [31]. First, 12 mL of 80% EtOH and 1.5 mL of zein solution were added to a glass bottle and stirred at 1000 rpm at 25 °C for 5 min. Then, 5 mL of TGP solution was added to the mixture and stirred for another 5 min. In addition, 3 mL γ-PGA was mixed with 28.5 mL ddH_2_O. Gradually, 31.5 mL of solution was added to form TGP_NP_ solution while stirring for 30 min. The mixture was then placed in a rotary evaporator to remove ethanol under reduced pressure in a 40 °C water bath. After ethanol removal, ddH_2_O was added to bring the total volume to 50 mL. Then, 100 µL of the sample was taken and diluted to 1 mL with ddH_2_O and then transferred to a dynamic-light-scattering (Zetasizer Nano ZS90, Malvern, UK) instrument to measure the particle size of the sample. The sample was then subjected to high-speed centrifugation at 12,000 rpm for 20 min to obtain the supernatant and pellet, which were analysed using HPLC to determine the encapsulation efficiency.

### 2.9. Estimating Encapsulation Efficiency of Nanoparticles

High-speed centrifugation was applied to determine whether nanoparticles were encapsulated; 1 mL of 300 μg/mL TGP_NP_ was added to centrifuge tubes and centrifuged at 10,000 rpm for 20 min. Following this, 900 μL of the supernatant was removed, and an equal volume of ethanol was added to redissolve the precipitated TGP_NP_. The precipitated portion was collected and redissolved in 80% ethanol to obtain a volume of 1 mL. The absorbance value was measured at UV 280 nm after the sample was processed by HPLC, and the obtained integrated area was used as the theoretical value. The freeze-dried TGP extract was dissolved in ethanol to prepare 300 μg/mL TGP_EtOH_ solution. After HPLC analysis, the encapsulation efficiency (EE) was calculated using the following formula:

EE = the integrated area representing TGP in TGP_NP_/the integrated area of TGP_EtOH_ before encapsulation × 100%

### 2.10. Statistical Analysis

For the in vitro experiments, Western blot analysis and a statistical one-way analysis of variance (ANOVA) were performed via the use of the software GraphPad Prism Ver 5.0 (GraphPad Software Inc., La Jolla, CA, USA), and *p*-values of <0.05 (*) were defined as statistically significant.

## 3. Results

### 3.1. TLC Analysis Revealed Similar Compositions in MTF and TGP

**Figure 1:**
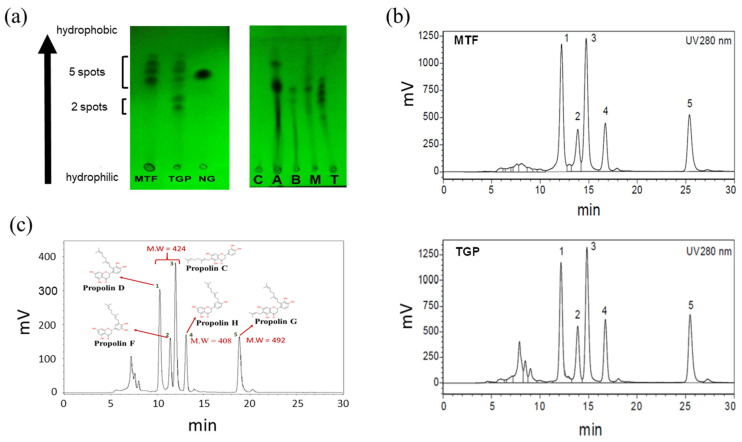
Component analysis of TGP and MTF. (**a**) Left panel: TLC analysis using the HEMe solvent revealed compositional differences among MTF, TGP, and the flavonoid standard, naringin (NG). Right panel: TLC analysis of the propolis samples from Canada (C), Australia (A), Brazil (B), Malaysia (M), and Taiwan (T). (**b**) HPLC analyses of MTF (upper panel) and TGP (lower panel) at a UV absorbance of 280 nm. (**c**) The molecular weights and chemical structures of the main components of TGP using LC/MS (appeared as the five peaks 1–5). Propolins C, D, and F with three different retention times (peaks 1–3) have an identical molecular weight (424 Da).

Previous research has found that TGP comes from *Macaranga tanarius* [12], so we wanted to confirm that the materials used in this experiment were the same. To analyse the compositions of MTF and TGP, thin-layer chromatography (TLC) was performed for their preliminary identification. This study is based on the difference in affinity toward the stationary phase to separate the components in propolis. TLC analysis can be used to examine propolis from around the world, and the chromatographic pattern can help determine the tested propolis’s origin. We utilised organic solvent mixtures with a volume ratio of n-hexane/ethyl acetate/methanol of 6:3:1, termed HEMe, as the elution solvent. The elution patterns of MTF and TGP were compared (Figure 1a, left panel), demonstrating that the molecule composition is similar in the two samples, except for the lower bands in TGP representing the hydroxylated flavonoids according to previous studies [23]. Moreover, we compared the compositions of propolis from different regions (Canada, Australia, Brazil, Malaysia, and Taiwan). The TLC analysis demonstrated distinct TLC band patterns (Figure 1a, right panel), implicating that the constituent profiles of propolis collected from different regions vary significantly.

### 3.2. HPLC Analysis of TGP and MTF

A previous study reported that the plant origin of TGP is MTF [20], a finding corroborated by Japanese researchers analysing green propolis and *Marcaranga tanarius* from the Okinawa region [12]. The plant’s flowers, fruits, leaves, and bark all contain a considerable amount of prenylflavonoids. The samples collected in the present study were from MTF harvested in Taiwan. The MTF was extracted with ethyl acetate, filtered, concentrated, dried, and then dissolved in methanol with a 2 mg/mL concentration for HPLC analysis. Initially, 87.5% methanol was used to examine the distribution of the constituent molecules of the mixture since the isomers separated at relatively close times (Supplementary Figure S2). Therefore, in the second analysis, the eluent was adjusted to a gradient of 85% to 90% methanol, resulting in a better separation of the components, with the molecular weight observed at a discrete retention time. Based on the HPLC absorbance profiles measured at 280 nm (Figure 1b), the main components of both TGP and MTF exhibited five major peaks with similar retention times. These results are consistent with those reported previously [16,33] and provide preliminary evidence demonstrating that the source of TGP is likely to be MTF.

### 3.3. Establishment of LC/MS Spectra for TGP Extract

The components of TGP were identified using LC/MS analysis. The operating conditions were the same as those used for HPLC. A methanol gradient was used as the eluent, and five separated peaks were analysed to obtain their molecular weights. By comparing the molecular weights with the organic compound database [12,33], the chemical structure of TGP was deduced, revealing consistency with previously reported chemical structures. As shown in Figure 1c, the molecular weights of the main components are 408 Da, 424 Da, and 492 Da, representing propolin H; propolins C, D, and F (isomeric compounds); and propolin G, respectively [34].

### 3.4. Cell Viability Characterisation After TGP_DMSO_ Treatment Using MTT

**Figure 2:**
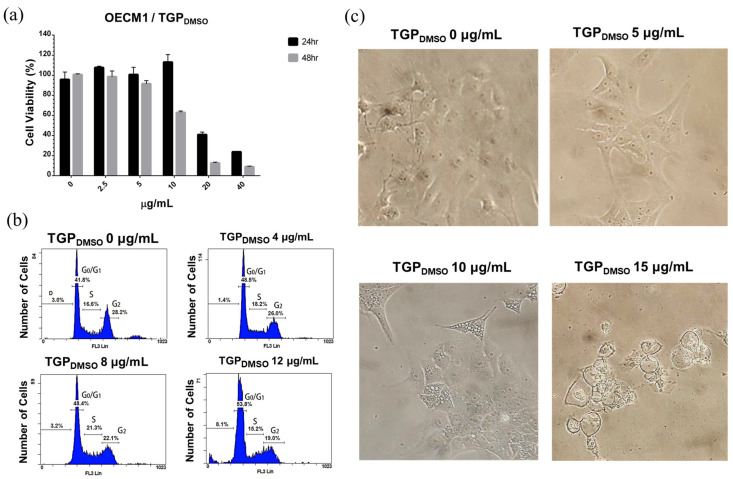
TGP_DMSO_ cytotoxicity analysis. (**a**) MTT assay of TGP_DMSO_-treated OECM1 cells shows a 24 h IC_50_ of 18 µg/mL and a 48 h IC_50_ value of 12.6 µg/mL. Data represent the mean ± SD from three independent experiments (*n* = 3). (**b**) Flow cytometry analysis shows a TGP_DMSO_ concentration-dependent effect on OECM1 growth arrest in the S phase. (**c**) TGP_DMSO_-induced changes in cell morphology.

To assess the potential of TGP_DMSO_ in cancer therapy, the Taiwanese OECM1 human oral squamous carcinoma cell line was used for cell viability analysis. As previous studies have demonstrated that propolis reveals cytotoxicity at IC_50_ of 20 µg/mL [16], the medium was added with TGP_DMSO_ at 0, 2.5, 5, 10, 20, and 40 µg/mL. Cell viability was monitored using an MTT assay after 24 and 48 h of culture (Figure 2a). The half-maximal inhibitory concentration (IC_50_) after 24 h was 18 µg/mL, and the IC_50_ after 48 h was 12.6 µg/mL, which is similar to the IC_50_ of 10 µg/mL reported previously when using the purified TGP component propolin F for 24 h [35].

### 3.5. Cell Cycle Analysis of OECM1 Cells in TGP_DMSO_-Containing Culture Medium

Cell cycle profiles were observed using flow cytometry to study the impact of TGP_DMSO_ in OECM1 cells. Once the cell growth stabilised, the culture medium was replaced with fresh culture medium containing 0, 4, 8, and 12 µg/mL of TGP_DMSO_. The cells were cultured for 48 h and then collected for PI staining. Notably, exposure to TGP_DMSO_ at a concentration of 8 µg/mL induced cell cycle arrest in the S phase, and the cell number in the S phase increased from 16.6% to 21.3% (Figure 2b). At a concentration of 12 µg/mL, the cell number in the G2 phase decreased from 28.2% to 26.0%, 22.1%, and 19.0%, showing signs of DNA fragmentation, which increased from 3.0%, 3.1%, and 1.4% to 8.1%. These results indicate that high concentrations of TGP_DMSO_ induce cell cycle arrest in cancer cells and may have a pro-apoptotic effect.

### 3.6. TGP_DMSO_ Induces Apoptosis and Cell Morphology Changes

TGP_DMSO_-induced cell morphology changes were also observed at different concentrations of TGP_DMSO_. The OECM1 cells were treated with TGP_DMSO_ at 0, 5, 10, and 15 µg/mL concentrations for 48 h; this was followed by morphological observation using microscopy (Nikon, TS100, Tokyo, Japan). At a TGP_DMSO_ concentration of 5 µg/mL, the original squamous morphology of the OECM1 cells became more spherical (Figure 2c, Supplementary Figure S3), exhibiting considerably fewer extensions than the control cells. Cell growth was slower at a TGP_DMSO_ concentration of 10 µg/mL, with many cells displaying a triangular shape with numerous small vesicles forming inside. At a TGP_DMSO_ concentration of 15 µg/mL, some cells exhibited a circular shape, with a few cells floating, indicating cell death.

### 3.7. Western Blotting Demonstrates That TGP_DMSO_ Facilitates Apoptosis in OECM1 Cells

**Figure 3:**
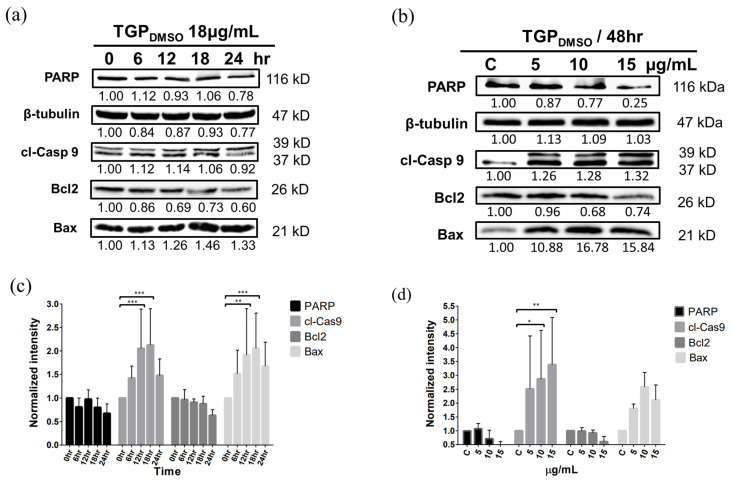
Western blot analysis indicates TGP_DMSO_-induced apoptosis in OECM1 cells. (**a**) Time-dependent (0–24 h) Western blot analysis of TGP_DMSO_-treated (18 µg/mL) OECM1. (**b**) Dose-dependent (0–15 µg/mL) Western blot analysis of TGP_DMSO_-treated OECM1 after 48 h. β-Tubulin was used as a loading control for protein quantification. (**c**,**d**) Quantitative analysis of apoptosis-related gene expression in (**a**) and (**b**), respectively, using ImageJ software (v1.54d, National Institutes of Health, USA). The expression levels for each protein were normalised using the cell sample harvested at 0 h in (**c**) and non-TGP_DMOS_-treated sample (0 µg/mL) in (**d**). Data represent the mean ± SD from three independent experiments (*n* = 3). Significantly different from control, * *p* < 0.05, ** *p* < 0.01, *** *p* < 0.001.

To inspect the apoptotic gene expression profile of OECM1 cells after TGP_DMSO_ treatment, the cells were treated with 18 µg/mL of TGP_DMSO_ and were collected every 6 h from 0 to 24 h for Western blot analysis (Figure 3a, Supplementary Figure S4 and Table S1). The expression profiles of apoptotic markers were also studied after 0, 5, 10, and 15 µg/mL of TGP_DMSO_ treatments (Figure 3b, Supplementary Figure S5 and Table S2). The apoptotic marker proteins involve cleaved caspase 9 (cl-Casp 9), Bax, Bcl2, and PARP with the benchmark protein β-tubulin. The experimental results demonstrated that 18 µg/mL TGP_DMSO_ induced apoptosis in the OECM1 cells (Figure 3c) with decreased PARP and Bcl-2 expression levels and increased cl-Casp 9 and Bax protein levels after 18 h of culture. However, 5 µg/mL of TGP_DMSO_ treatment can induce the apoptosis of OECM1 cells after 48 h (Figure 3d).

### 3.8. Preparation and Analysis of Nanoencapsulated TGP_NP_

**Figure 4:**
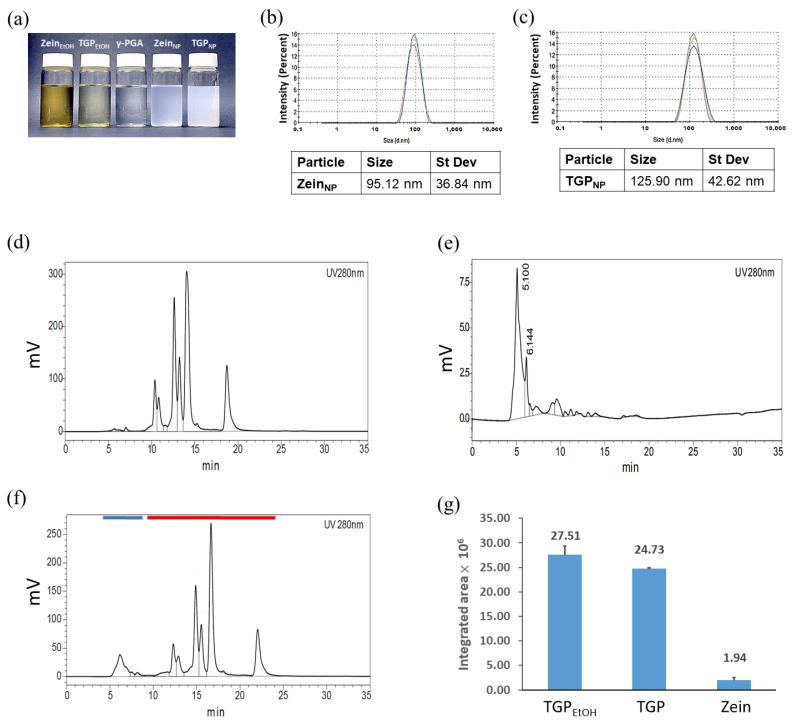
Qualitative analysis of TGP_NP_. (**a**) Comparison of the appearance of solutions of zein and TGP dissolved in EtOH (Zein_EtOH_ and TGP_EtOH_, respectively), γ-PGA, zein nanoparticles (Zein_NP_), and TGP_NP_. (**b**,**c**) DLS analysis of Zein_NP_ and TGP_NP_ with average sizes of 95.12 nm and 25.9 nm, respectively (*n* = 10). Each repeat is shown in different colours. (**d**–**f**) HPLC profiles of (**d**) TGP_EtOH,_ (**e**) Zein_NP_, and (**f**) TGP_NP_. (**g**) Encapsulation efficiency of TGP in zein nanoparticles analysed by the HPLC profiles. TGP_EtOH_: The integrated area of HPLC peaks for TGP_EtOH_ from (**d**). TGP: The integrated area of HPLC peaks for TGP (red line) from (**f**), representing the TGP content in TGP_NP_. Zein: The integrated area of HPLC peaks for zein (blue line) from (**f**), representing the zein content in TGP_NP_. Data represent the mean ± SD from three independent experiments (*n* = 3).

The aforementioned experiments used DMSO as the solvent for TGP sample preparation. To avoid the cytotoxicity of DMSO (Supplementary Figure S6), we aimed to examine the bioactivity of TGP encapsulated in zein nanoparticles. In this study, food-grade zein and γ-PGA were used for TGP nanoencapsulation (TGP_NP_) [31]. First, TGP and zein were separately dissolved in 80% ethanol; they were then added to γ-PGA aqueous solution for self-encapsulation. Subsequently, ethanol was removed under reduced pressure, and an appropriate amount of ddH_2_O was added to obtain the required volume and concentration for the TGP_NP_. During preparation, as the proportion of γ-PGA added increased, the ethanol solution of TGP_NP_ became increasingly opaque from the original transparent state, indicating successful drug encapsulation (Figure 4a). If the solution turns milky during preparation, this indicates that the particle size is too large, requiring readjustment of the stirring speed and ambient temperature. The samples were analysed using dynamic light scattering (DLS) to determine the particle size. The results revealed that the average size of unloaded zein nanoparticles (Zein_NP_) was 95.12 nm (Figure 4b), whereas TGP_NP_ showed an average size of 125.9 nm (Figure 4c). In terms of practical applications, the particle size of TGP_NP_ is suitable for cellular uptake and can be directly absorbed by cells [32].

We performed HPLC to calculate the encapsulation efficiency (EE) of TGP in zein nanoparticles, with the absorbance measured at UV 280 nm. The HPLC profiles of TGP_EtOH_ 300 µg/mL and zein_EtOH_ solutions were collected individually, as shown in Figure 4d and Figure 4e, respectively. For the TGP_NP_ sample, the HPLC peaks representing zein (blue line in Figure 4f) and TGP_NP_ (red line in Figure 4f) were well resolved using HPLC. The encapsulation efficiency of TGP in zein nanoparticles was calculated as 82.83% (Figure 4g) (see Section 2 and Supplementary Table S3 for details).

### 3.9. TGP_NP_ Retain Cytotoxicity Comparable to OECM1

**Figure 5:**
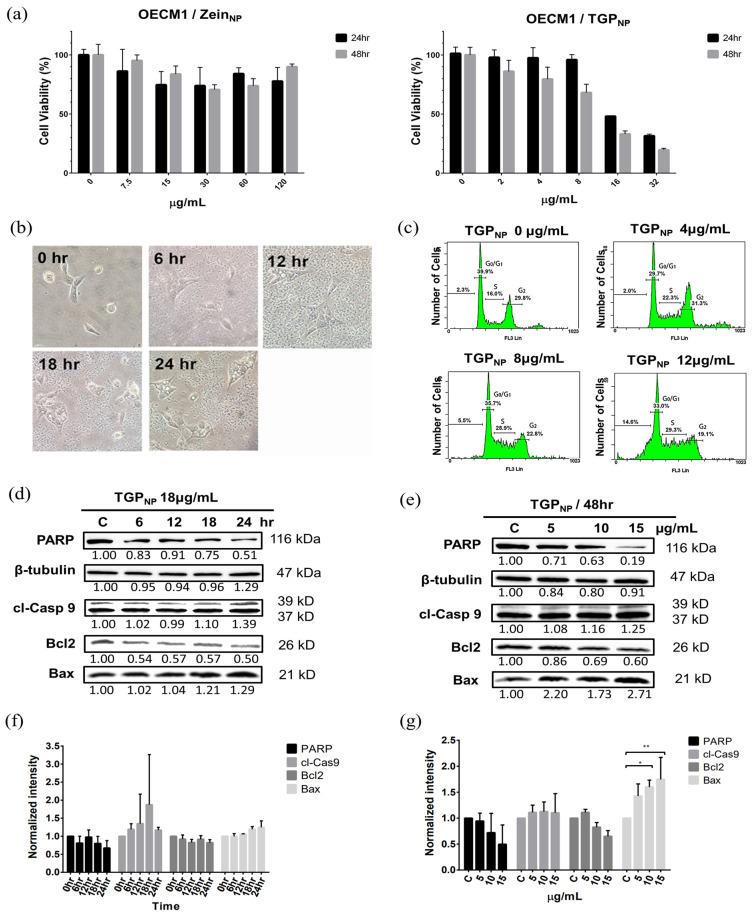
Western blot analysis indicates TGP_NP_-induced apoptosis in OECM1 cells. (**a**) MTT assays using OECM1 cells after Zein_NP_ (left panel) and TGP_NP_ (right panel) treatment for 24 h and 48 h. IC_50_ of TGP_NP_ was determined using the OECM1 cell viability after 48 h treatment of TGP_NP_. (**b**) The morphological images of OECM1 cells treated with TGP_NP_ (18 µg/mL) at every 6 h within 24 h. (**c**) Flow cytometry analysis of cell cycle in OECM1 cells treated with varying concentrations of TGP_NP_. (**d**) Time-dependent Western blot analysis of TGP_NP_ treatment at 18 µg/mL. (**e**) Dose-dependent Western blot analysis after 48 h of TGP_NP_ treatment. β-Tubulin was used as a loading control for protein quantification. (**f**,**g**) Quantitative analysis of apoptosis-related gene expression in (**d**) and (**e**), respectively, using ImageJ software. The expression levels for each protein were normalised using the cell sample harvested at 0 h in (**f**) and non-TGP_DMOS_-treated sample (0 µg/mL) in (**g**). Data are presented as mean ± SD from three independent experiments. Significantly different from control, * *p* < 0.05, ** *p* < 0.01.

To characterise the OECM1 cytotoxicity of TGP_NP_, various concentrations of TGP_NP_ were added to the cells, ranging from 0 to 32 µg/mL. The empty Zein_NP_ show moderate toxicity to OECM1 (Figure 5a, left panel). MTT assays were performed after 48 h of incubation. The results showed that, at 4 µg/mL of TGP_NP_, the cell viability was approximately 80%, and at 16 µg/mL, the cell viability was approximately 35%. The IC_50_ of TGP_NP_ was 11.6 µg/mL (Figure 5a, right panel). The value was very similar to TGP_DMSO_ (Figure 2a), demonstrating that the TGP_NP_ could be taken up by cells to induce cytotoxicity.

### 3.10. Apoptosis and Morphological Changes in OECM1 Cells After Uptake of TGP_NP_

To understand the anticancer effects of TGP_NP_, the TGP_NP_ concentration of 18 µg/mL was applied after the MTT assay. The experimental results revealed that, at 0 h, the OECM1 cells were elongated and squamous. After 6 h, the cells became triangular with small vesicles formed within, and nanoparticle aggregation and sedimentation were observed (Figure 5b, Supplementary Figure S7). After 12 h, the cells appeared to have a triangular morphology with large vesicles. By 18 and 24 h, vesicles of various sizes developed in the cells, with some cells showing spherical morphology and losing dish-attaching ability, indicating that TGP_NP_ treatment could induce OECM1 apoptosis.

### 3.11. TGP_NP_ Arrested the Cell Cycle and Induced the Apoptotic Protein Expression of OECM1 Cells

Previously, DMSO was used as the solvent of TGP to perform cytotoxicity characterisation, demonstrating that TGP_DMSO_ caused cell cycle arrest in the S phase and induced apoptosis. We were eager to compare the effectiveness of TGP_NP_’s anticancer activity. TGP_NP_ encapsulated in nanoparticles was added to culture dishes at concentrations of 0, 4, 8, and 12 µg/mL. After 48 h, OECM1 cells were collected, stained, and analysed using flow cytometry to observe cell changes. At a TGP_NP_ concentration of 0 µg/mL, 16.0% of the cells underwent arrest in the S phase (Figure 5c). When the TGP_NP_ concentration increased to 4 and 8 µg/mL, 22.3% and 28.9% of the cells underwent arrest in the S phase, respectively. At a TGP_NP_ concentration of 12 µg/mL, 29.3% of cells underwent arrest in the S phase, and DNA fragmentation increased from 2.3%, 2.0%, and 5.5% to 14.6%. Western blot analysis of apoptosis-related proteins revealed that the expression of cl-Casp 9 and Bax increased with increasing TGP_NP_ concentrations, whereas the expression of Bcl2 and PARP decreased with increasing TGP_NP_ concentrations (Figure 5d–g, Supplementary Figures S8 and S9, and Supplementary Tables S4 and S5). These results provided evidence that TGP_NP_ promotes the apoptosis of OECM1 cells.

## 4. Discussion

### 4.1. The Ingredients of Taiwan Green Propolis Are Unique Due to the Habits of Bees and Taiwan’s Geographical Environment

Taiwan features a unique geographical and ecological environment. Within 40,000 square kilometres, Taiwan encompasses tropical, subtropical, temperate, and subarctic climates, creating a highly diverse ecosystem of plants. It is well known that the composition of propolis is closely tied to the plant species in the areas where bees are active [6]. According to the current literature, the only bees in Taiwan known to generate propolis are *Lepidotrigona hoozana* (a stingless bee) and commercially farmed honeybees (*Apis mellifera*) [36]. *L. hoozana* is active in mid- to low-altitude areas and has not yet been commercially cultivated, and the composition of its propolis sources remains unclear. Honeybees, on the other hand, are extensively bred and commonly collect nectar and resins in low-altitude and flatland areas. Analysis reveals that the composition of green propolis generated in spring and summer is highly similar [37].

This study confirms that the source of TGP propolis is the fruit peel of *Macaranga tanarius*, while previous research has shown that BGP originates from the buds of *Baccharis dracunculifolia* [11]. The differences between the source plants and components result in significant compositional differences between these two types of green propolis. One of the main advantages of consuming fresh propolis is that bees collect fresh resin directly from plants, which retains a rich amount of effective components from the resins while removing the nonfunctional substances. As a result, propolis has relatively high levels of flavonoids and polyphenols and a lower complexity of non-essential compounds.

### 4.2. Rapid Testing of TGP Source Through TLC

Previous studies have shown that propolis has anticancer and health-promoting functions [5]. However, the composition of propolis collected from different parts of the world varies, primarily due to the types of bees and the local plants involved. Despite advances in modern technologies and analytical capabilities, controlling bees’ foraging behaviour is still impossible. Therefore, propolis is mainly classified based on season and regionality. Currently, the quality of propolis is mainly assessed and graded on the basis of the contents of flavonoids and phenolic compounds.

In order to characterise the regionality and source of the propolis, developing an easy and economical methodology is essential in the industry. Conventionally, LC/MS and HPLC analyses were used for raw material accreditation and quality control [38]. In this study, component analysis using TLC with the solvent system HEMe provides an efficient assay for profiling the ingredients in the propolis and its relationship with the surrounding plants. The methodology utilised to analyse propolis from various countries revealed considerable differences in composition. In the future, constructing an online database of propolis from different origins would enable a straightforward comparison and differentiation. The database would serve as a hub providing data on the composition and differentiating propolis products worldwide.

### 4.3. TGP Crude Extracts and Purified Products Exhibit the Same Cytotoxicity for Cancer Cells

Research has reported that the secondary metabolites of plants, namely flavonoids, have multifunctional properties [2]. Flavonoids scavenge free radicals to achieve antioxidant effects and exhibit antiviral and antibacterial properties [39]. Additionally, flavonoids also repair cells and have growth-hormone-like characteristics [40]. Most valuably, flavonoids cause the apoptosis of cancer cells [39]. Recent research has indicated that, compared with unprenylated flavonoids, prenylated flavonoids have higher selective cytotoxicity against cancer cells [40]. Propolin C exhibits a strong antioxidant and anti-inflammatory activity, scavenging free radicals [14]. The TGP_DMSO_ extract used in this study inhibited the growth of cancer cells, with an IC_50_ of approximately 18 µg/mL at 24 h after treatment. This value is comparable to the IC_50_ of 20 µg/mL (8.5 µM) purified propolin C, a component of TGP [41]. In addition, compared with the purified product Artepillin C, a major component of BGP, whose IC_50_ was 100 µg/mL [42]. Reviewing previous research reports, we found that the cytotoxicity analysis of using clinical chemotherapy drugs paclitaxel and cisplatin on OECM1 showed that their IC_50_ values for 48 h were 11.7 and 228.3 µg/mL [43]. Compared with cisplatin, TGP has better cancer cytotoxicity. BGP is the most extensively studied propolis, with a flavonoid content of 2.86% (*w*/*w*) and a phenolic compound content of 37.8% (*w*/*w*) [38]. However, it has poor thermal stability and is easily degraded in an environment above 50 °C [44]. In contrast, TGP composition is relatively simple, and even the unrefined crude extract contains more than 65% flavonoid [33], and TGP still remains stable at temperatures of up to 120 °C [45]. Moreover, compared with the well-studied flavonoid compound, luteolin, which is currently being studied in nanoparticle form for anticancer applications [46,47], TGP consisting of the flavonoids with prenyl group modifications is three-fold more cytotoxic than luteolin [41,48]. Furthermore, the encapsulation efficiency in this study reached up to 83%, higher than the PPS-PEG nanoparticle encapsulation efficiency of luteolin [47].

### 4.4. TGP_NP_ Can Achieve Anticancer Efficacy Comparable to DMSO-Dissolved TGP

Research has indicated that TGP exerts exceptional cytotoxic effects on liver, lung, brain, and melanoma cancer cells. In this study, oral cancer cells were utilised, providing a cell model to evaluate the antitumour effectiveness of oral cancer through oral administration. TGP_DMSO_ reveals an inhibitory effect on OECM1 cells; however, DMSO-induced cytotoxicity due to cell membrane disruption and protein denaturation may raise safety concerns. For TGP_NP_, an IC_50_ value of 11.6 µg/mL was attained, similar to TGP_DMSO_. According to the flow cytometry results, the TGP_NP_ concentration of >4 µg/mL promoted cell arrest in the S phase, and when the TGP_NP_ concentration was increased to >12 µg/mL, the proportion of apoptotic cells considerably increased. Through the observation of cell morphology and the Western blot analysis of apoptosis-related proteins, this study demonstrated that the levels of Bax and cl-Casp 9 were increased with the increase in TGP_NP_ concentration, whereas the levels of Bcl2 and PARP were decreased, suggesting that OECM1 cells undergo an intrinsic apoptosis pathway [49]. Yet, the detailed molecular mechanism by which TGP triggers Bax activation and the subsequent downstream cascade remains to be fully elucidated, requiring further experimental investigation and potentially AlphaFold predictions [25].

### 4.5. TGP_NP_ Demonstrate Excellent Safety and Multiple Advantages

Nanotechnology has rapidly developed in recent years. The primary objective is to enhance cellular uptake, which can be achieved by manipulating chemical characteristics, such as particle size, charge, shape, and material properties. Various materials, such as metal particles [50], phospholipids [24], lipoproteins, and mixtures of zein and γ-PGA, have been developed. The methods employed for nanoencapsulation include emulsification, spray-drying, and phase transition, each with its unique features and technical limitations. Zein, which is extracted from maize, offers multiple advantages for applications. For example, the solvent used in the experimental process is ethanol, which can be removed under reduced pressure, thereby avoiding the risk of toxicity associated with organic solvents [51]. Previous research utilised zein nanoparticles in food-grade pharmaceuticals, such as curcumin [52].

Since the isoelectric point of zein is at pH 6.2 [31], zein tends to precipitate in this pH environment. In addition, the flavonoids in TGP change colour in alkaline solutions. Therefore, zein nanoparticles are expected to be relatively stable in a pH range between 6.2 and 7.4. During the fabrication process, zein nanoparticles can form stably at temperatures between 25 °C and 45 °C. As such, under the physiological pH values and temperatures, TGP_NP_ are considered to be in a stable state. A very small number of people may have allergic reactions to propolis, which is related to each individual’s level of immune system activation. To avoid allergic responses, it is recommended to start with a small amount of propolis and gradually increase the dosage after the body has adapted until reaching the normal intake level.

The experimental results of this study may shed light on the therapeutic development of food-grade TGP nanoparticles using zein and γ-PGA as a cure for oral cancer. This study demonstrates that the cytotoxicity of TGP_NP_ against OECM1 cells is comparable to that of TGP_DMSO_. However, to gain a deeper understanding of its biosafety and bioavailability, evaluating its anticancer efficacy in animal models is crucial. Additionally, assessing its physiological effects is essential to preventing non-specific cytotoxicity.

## 5. Conclusions

This study identified five types of prenylflavonoids from TGP using HPLC and LC/MS, indicating that the components of TGP correlate with the plant near the habitat of Taiwanese bees. While the TGP substances were not individually purified, the crude extract of TGP exhibits anticancer efficacy comparable to that of purified substances, demonstrating its potential as an anticancer reagent. In this study, food-grade zein and γ-PGA were used to prepare TGP_NP_ with a particle size of 110 nm, exhibiting similar anticancer activity compared to TGP_DMSO_ while offering advantages in biosafety and reduced cytotoxicity. The TGP_NP_-induced apoptosis in OECM1 cells highlights its capacity and feasibility in the development of oral cancer treatments.

## Data Availability

The original contributions presented in this study are included in this article. Further enquiries can be directed to the corresponding author.

## Supplementary Materials

The following supporting information can be downloaded at https://www.mdpi.com/article/10.3390/biomedicines13040921/s1, Figure S1: The original PVDF transfer membrane of Western blot analysis of OECM1 cells treated with TGP_NP_ shows the time- and dose-dependent effects. The membranes were cut into 5 pieces. The chemiluminescent and visible light merged diagram was visualized by the secondary antibodies of the apoptosis-related proteins; Figure S2: The HPLC analysis of TGP. 87.5% methanol was used as the mobile phase to investigate the compositional distribution of the mixture; Figure S3: The morphology of OECM1 cells treated with different concentrations of TGP_DMSO_. Top panel shows the original microscopic view. The red boxes indicate the magnified regions shown in the bottom panel; Figure S4: The triplicate of the time-dependent Western blot analysis after 0, 6, 12, 18 and 24 h of 18 µg/mL TGP_DMSO_ treatment. β-tubulin was used as a loading control for protein quantification. The expression level of proteins was quantitatively analyzed using ImageJ. The data are presented as the mean ± SD from three independent experiments (*n* = 3); Figure S5: The triplicate of the dose-dependent Western blot analysis of OECM1 cells after 48 h of TGP_DMSO_ treatment. β-actin and β-tubulin were used as loading controls for protein quantification. The expression level of proteins was quantitatively analyzed using ImageJ. The data are presented as the mean ± SD from three independent experiments (*n* = 3); Figure S6: Cytotoxicity assay of DMSO against OECM1 cells. The MTT assay of OECM1 cells reveals IC_50_ values of 28.2 mg/mL 25.0 mg/mL after 24 and 48 h treatments. Three independent experiments were performed, and the data are presented as the mean ± SD (*n* = 4); Figure S7: The morphology of OECM1 cells treated with 18 μg/mL TGP_NP_ at different time points. Top panel shows the original microscopic view. The red boxes indicate the magnified regions shown in the bottom panel; Figure S8: The triplicate of the time-dependent Western blot analysis after 0, 6, 12, 18 and 24 h of 18 µg/mL for TGP_NP_ treatment. β-actin and β-tubulin were used as a loading control for protein quantification. Western blot analysis of TGP_NP_ was performed, followed by quantitative analysis using ImageJ. The data are presented as the mean ± SD from three independent experiments (*n* = 3); Figure S9: The triplicate of the dose-dependent Western blot analysis after 48 h of 0, 5, 10 and 15 µg/mL TGP_NP_ treatment. β-tubulin were used as a loading control for protein quantification. Western blot analysis of TGP_NP_ was performed, followed by quantitative analysis using ImageJ. The data are presented as the mean ± SD from three independent experiments (*n* = 3); Table S1: Quantitative analysis of the Western blot results of time-dependent effect of TGP_DMSO_. The data are presented as the mean ± SD from three independent experiments (*n* = 3); Table S2: Quantitative analysis of the Western blot results of the dose-dependent effects of of TGP_DMSO_ after 48 h treatment. β-Tubulin was used as a loading control for protein quantification (*n* = 3); Table S3: The encapsulation efficiency of TGP in zein nanoparticles; Table S4: Quantitative analysis of the Western blot results of the time-dependent effect of TGP_NP_ after 0, 6, 12, 18 and 24 h of treatment. β-Tubulin was used as a loading control for protein quantification (*n* = 3); Table S5: Quantitative analysis of the Western blot results of the dose-dependent of TGP_NP_ after 48 h treatment. β-Tubulin was used as a loading control for protein quantification (*n* = 3).

## Abbreviations

BGP	Brazilian green propolis
BSA	Bovine serum albumin
DLS	Dynamic light scattering
DMSO	Dimethyl sulfoxide
EE	Encapsulation efficiency
EtOH	Ethanol
γ-PGA	Gamma-polyglutamic acid
HEMe	n-Hexane/ethyl acetate/methanol, 6:3:1
LC/MS	Liquid chromatography/mass spectrometry
MDR	Multidrug resistance
MRP	Multidrug resistance protein
MTF	Fruit peel of *Macaranga tanarius*
MTT	3-[4,5-Dimethylthiazol-2-yl]-2,5 diphenyl tetrazolium bromide
PBS	Phosphate-buffered saline
PI	Propidium iodide
RIPA	Radioimmunoprecipitation assay buffer
TGP	Taiwan green propolis
TGP_DMSO_	TGP in DMSO
TGP_EtOH_	TGP in 80% ethanol stock solution
TGP_NP_	TGP nanoparticles
TLC	Thin-layer chromatography
