# Supplementary material for: Taiwan Green Propolis Nanoparticles Induce Antiproliferation and Apoptosis in Oral Cancer Cells
Source: Biomedicines. 2025 Apr 9;13(4):921. doi: 10.3390/biomedicines13040921 (PMC12025001; doi:10.3390/biomedicines13040921)
Supplement: Supplementary file 1 [file biomedicines-13-00921-s001.zip › biomedicines-3498998-supplementary.pdf]

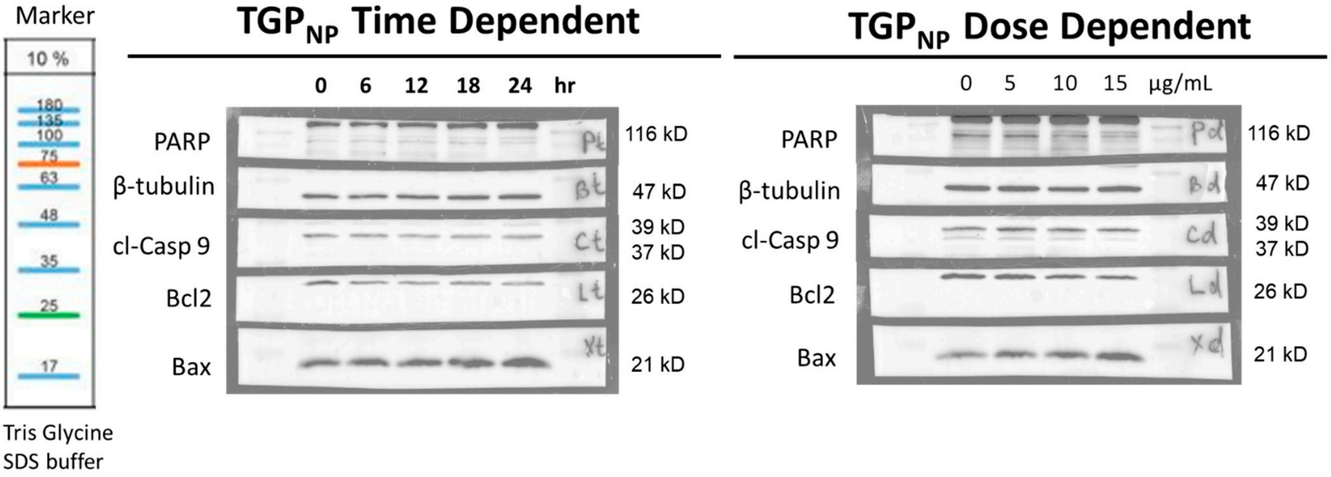

Supplementary Figure S1. The original PVDF transfer membrane of Western blot analysis of OECM1 cells treated with TGP_{NP} shows the time- and dose-dependent effects. The membranes were cut into 5 pieces. The chemiluminescent and visible light merged diagram was visualized by the secondary antibodies of the apoptosis-related proteins.

Analysis of TGP Composition by HPLC

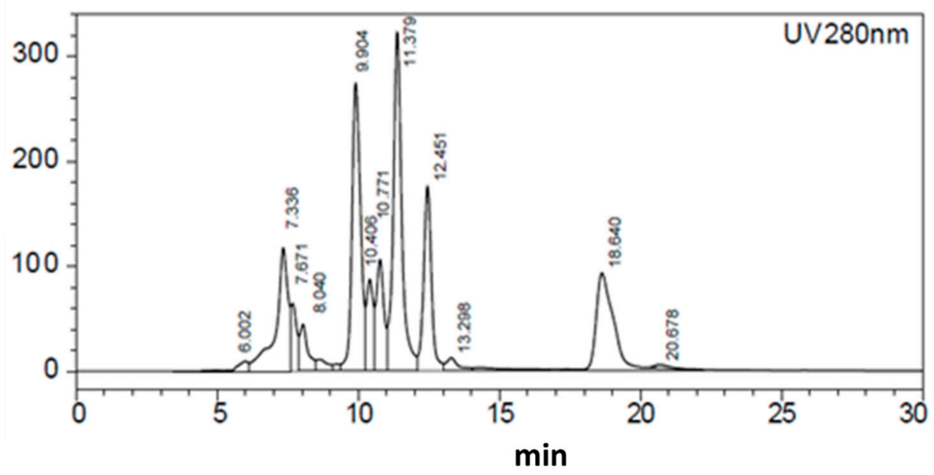

Supplementary Figure S2. The HPLC analysis of TGP. 87.5% methanol was used as the mobile phase to investigate the compositional distribution of the mixture.

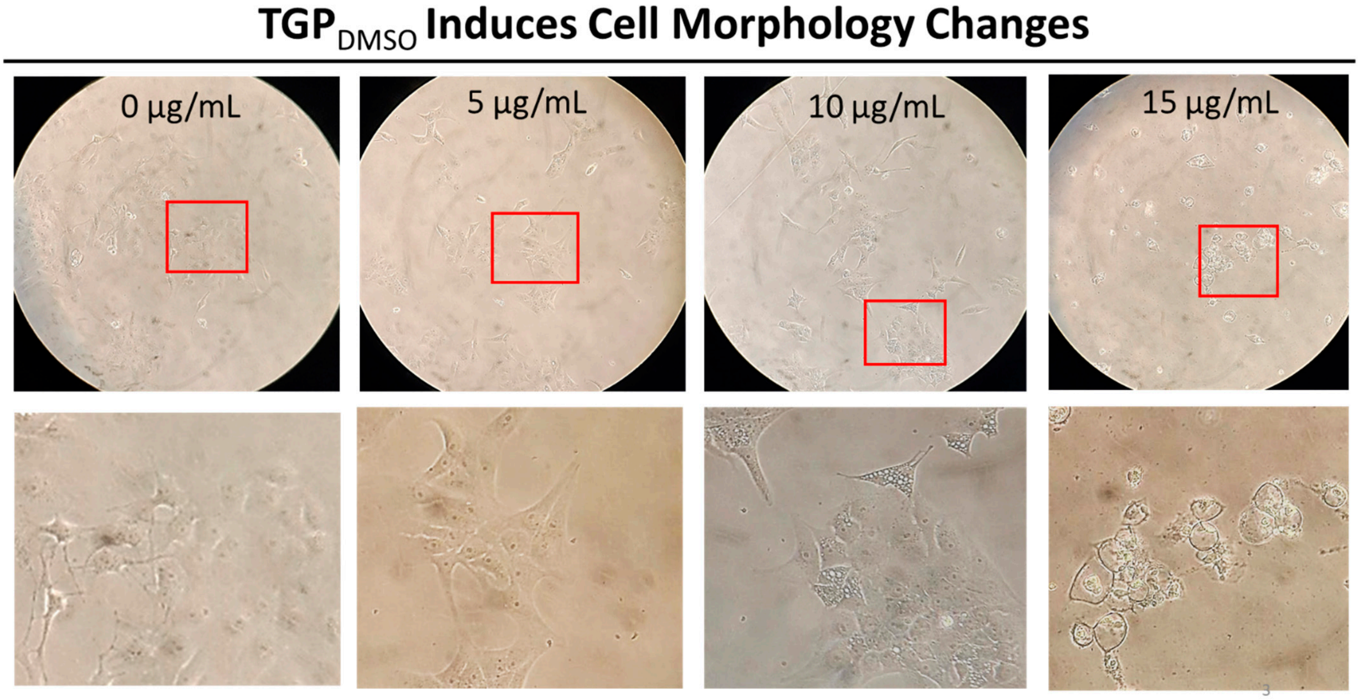

Supplementary Figure S3. The morphology of OECM1 cells treated with different concentrations of TGP$_{DMSO}$. Top panel shows the original microscopic view. The red boxes indicate the magnified regions shown in the bottom panel.

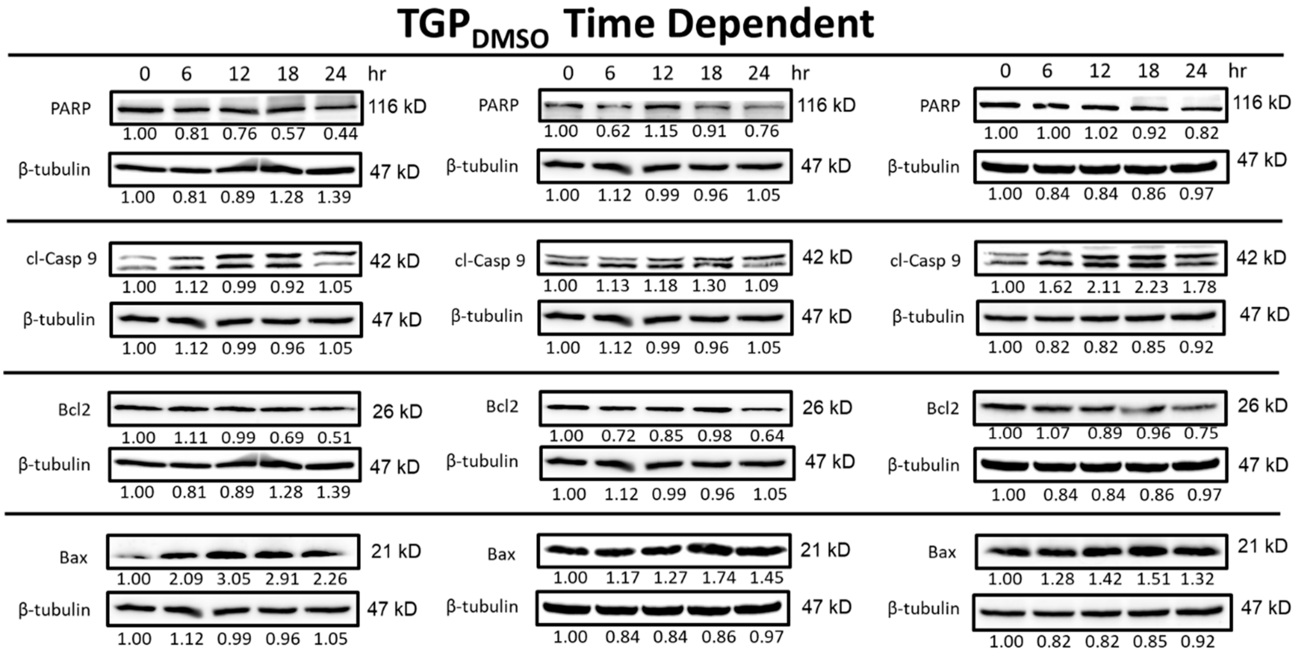

Supplementary Figure S4. The triplicate of the time-dependent Western blot analysis after 0, 6, 12, 18 and 24 hr of 18 µg/mL TGP_{DMSO} treatment. β-tubulin was used as a loading control for protein quantification. The expression level of proteins was quantitatively analyzed using ImageJ. The data are presented as the mean ± SD from three independent experiments (*n* = 3).

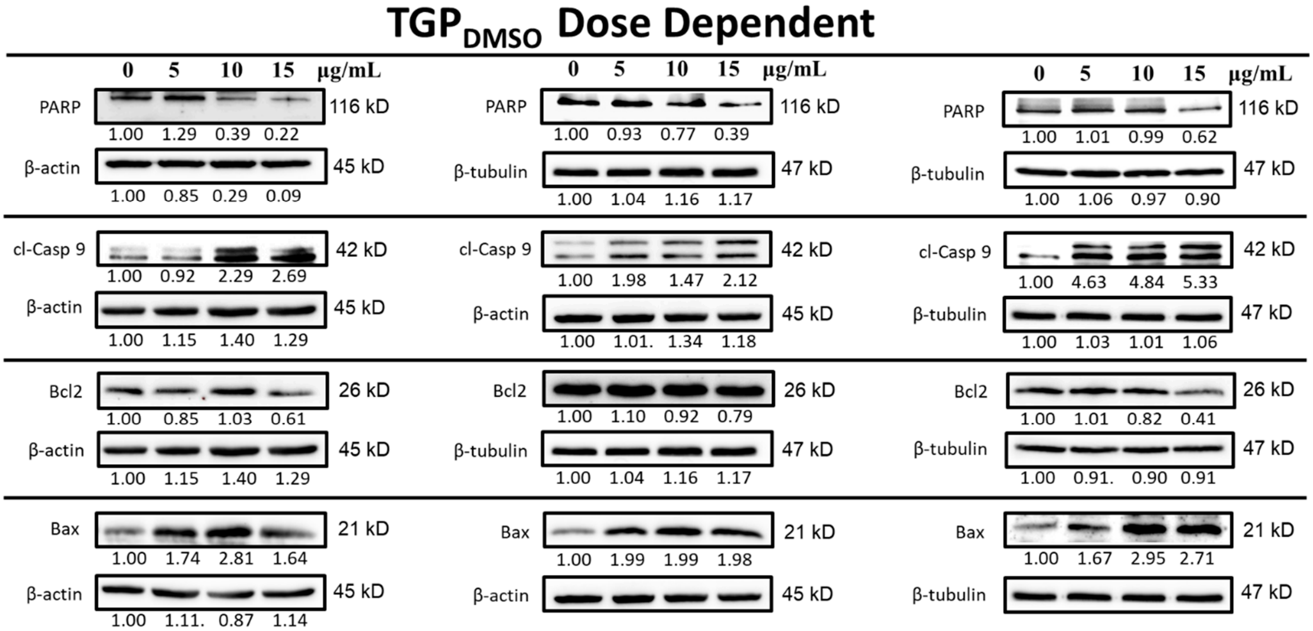

Supplementary Figure S5. The triplicate of the dose-dependent Western blot analysis of OECM1 cells after 48 hr of TGP_{DMSO} treatment. β-actin and β-tubulin were used as loading controls for protein quantification. The expression level of proteins was quantitatively analyzed using ImageJ. The data are presented as the mean ± SD from three independent experiments (*n* = 3).

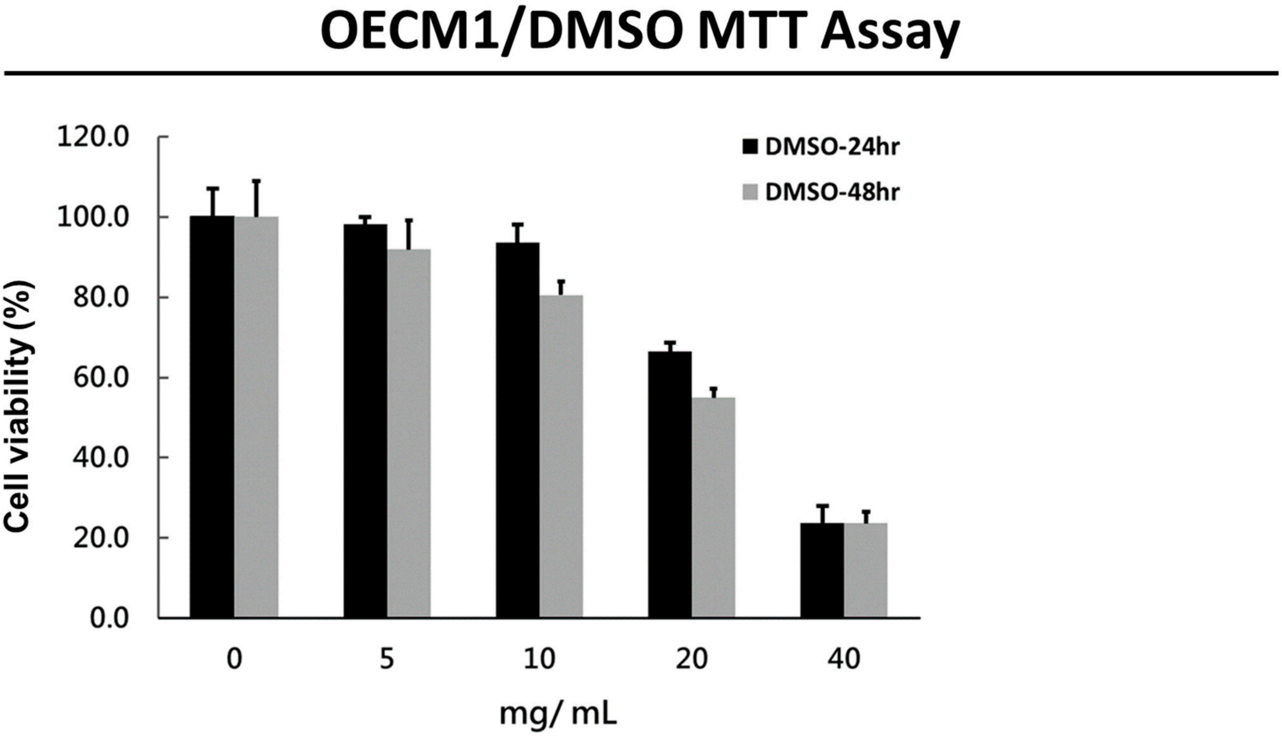

Supplementary Figure S6. Cytotoxicity assay of DMSO against OECM1 cells. The MTT assay of OECM1 cells reveals IC_{50} values of 28.2 mg/mL 25.0 mg/mL after 24 and 48 hr treatments. Three independent experiments were performed, and the data are presented as the mean ± SD (n = 4).

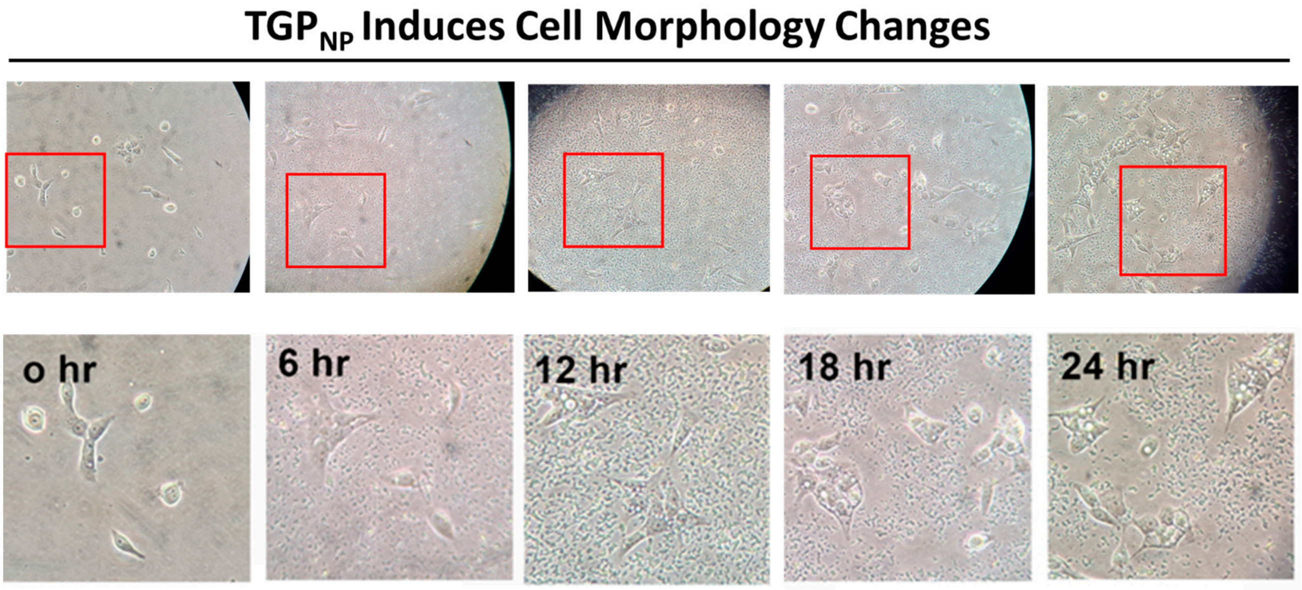

Supplementary Figure S7. The morphology of OECM1 cells treated with 18 µg/ml TGP_{NP} at different time points. Top panel shows the original microscopic view. The red boxes indicate the magnified regions shown in the bottom panel.

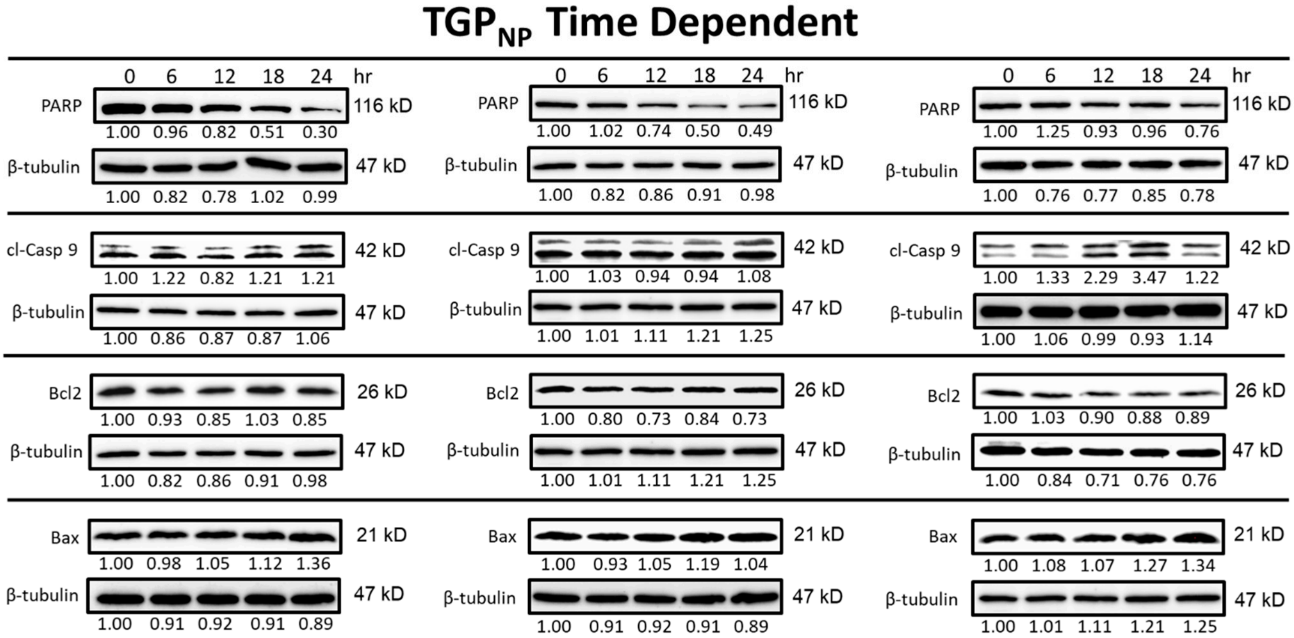

Supplementary Figure S8. The triplicate of the time-dependent Western blot analysis after 0, 6, 12 ,18 and 24 hr of 18 µg/mL for TGP$_{NP}$ treatment. β-actin and β-tubulin were used as a loading control for protein quantification. Western blot analysis of TGP$_{NP}$ was performed, followed by quantitative analysis using ImageJ. The data are presented as the mean ± SD from three independent experiments (n = 3).

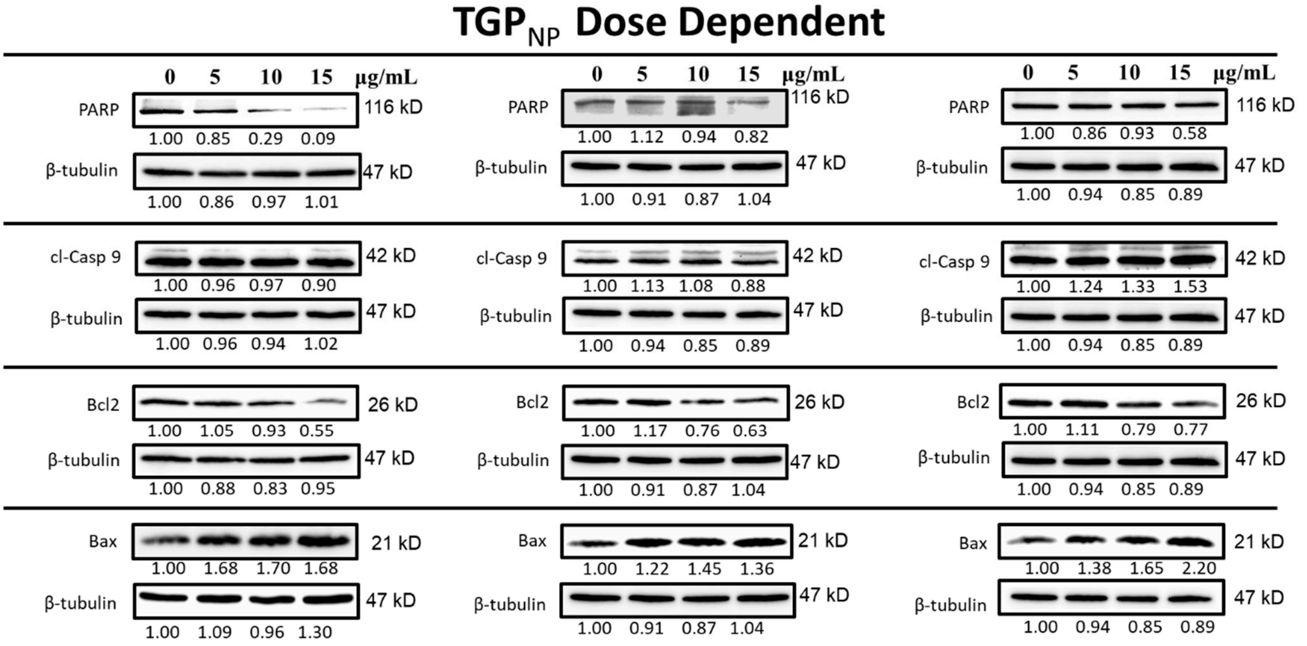

Supplementary Figure S9. The triplicate of the dose-dependent Western blot analysis after 48 hr of 0, 5, 10 and 15 μg/mL TGP_{NP} treatment. β-tubulin were used as a loading control for protein quantification. Western blot analysis of TGP_{NP} was performed, followed by quantitative analysis using ImageJ. The data are presented as the mean ± SD from three independent experiments (*n* = 3).

Supplementary Table S1. Quantitative analysis of the Western blot results of time-dependent effect of TGP_{DMSO}. The data are presented as the mean ± SD from three independent experiments (*n* = 3).

TGP_{DMSO} Time Dependent

Protein	Time	SUM	STDEV
**PARP**	0hr	1.00	0.00
	6hr	0.81	0.15
	12hr	0.98	0.14
	18hr	0.80	0.21
	24hr	0.67	0.31
**Casp-9**	0hr	1.00	0.00
	6hr	1.42	0.26
	12hr	2.05	0.84
	18hr	2.12	0.78
	24hr	1.48	0.35
**Bcl2**	0hr	1.00	0.00
	6hr	0.96	0.21
	12hr	0.91	0.07
	18hr	0.88	0.17
	24hr	0.63	0.12
**Bax**	0hr	1.00	0.00
	6hr	1.52	0.50
	12hr	1.91	0.99
	18hr	2.05	0.75
	24hr	1.68	0.51

Supplementary Table S2. Quantitative analysis of the Western blot results of the dose-dependent effects of of TGP_{DMSO} after 48 hrs treatment. β-Tubulin was used as a loading control for protein quantification (*n* = 3).

TGP_{DMSO} Dose Dependent

Protein	Dose	SUM	STDEV
**PARP**	0 μg/mL	1.00	0.00
	5 μg/mL	1.08	0.19
	10 μg/mL	0.72	0.30
	15 μg/mL	0.41	0.20
**Casp-9**	0 μg/mL	1.00	0.00
	5 μg/mL	2.51	1.91
	10 μg/mL	2.87	1.76
	15 μg/mL	3.38	1.71
**Bcl2**	0 μg/mL	1.00	0.00
	5 μg/mL	0.99	0.13
	10 μg/mL	0.92	0.10
	15 μg/mL	0.60	0.19
**Bax**	0 μg/mL	1.00	0.00
	5 μg/mL	1.80	0.17
	10 μg/mL	2.58	0.52
	15 μg/mL	2.11	0.55

Supplementary Table S3. The encapsulation efficiency of TGP in zein nanoparticles.

**TGP_{EtOH} Sample**	**TGP_{EtOH} Area**
TGP01	25848497
TGP02	29485570
TGP03	27194152
**Average**	**27509406**
**STDEV**	1838917

**TGP_{NP} Sample**	**Total Area**	**Zein** Area**	**TGP* Area**
TGP_{NP}0422	24680460	1928240	22752220
TGP_{NP}0423	25135632	2509914	22625718
TGP_{NP}0414	24363890	1380444	22983446
**Average**	**24726661**	**1939533**	**22787128**
**STDEV**	387940	564820	181401

EE = the amount of TGP (area) in NPs /the amount of TGP (area) before encapsulation × 100%

= $(TGP^*-Zein^{**})/TGP_{EtOH} \times 100\%$

= $(24726661-1939533) / 27509406 \times 100\%$

= $82.83 \pm 1.15\ \%$

Supplementary Table S4. Quantitative analysis of the Western blot results of the time-dependent effect of TGP_{NP} after 0, 6, 12, 18 and 24 hrs of treatment. β-Tubulin was used as a loading control for protein quantification ($n = 3$).

TGP_{NP} Time Dependent

Protein	Time	SUM	STDEV
	0hr	1.00	0.00
	6hr	1.08	0.15
**PARP**	12hr	0.83	0.10
	18hr	0.66	0.26
	24hr	0.52	0.23
	0hr	1.00	0.00
	6hr	1.19	0.15
**Casp-9**	12hr	1.35	0.82
	18hr	1.87	1.39
	24hr	1.17	0.08
	0hr	1.00	0.00
	6hr	0.92	0.12
**Bcl2**	12hr	0.83	0.09
	18hr	0.92	0.10
	24hr	0.82	0.08
	0hr	1.00	0.00
	6hr	1.00	0.08
**Bax**	12hr	1.06	0.01
	18hr	1.19	0.07
	24hr	1.25	0.18

Supplementary Table S5. Quantitative analysis of the Western blot results of the dose-dependent of TGP_{NP} after 48 hrs treatment. β-Tubulin was used as a loading control for protein quantification (*n* = 3).

TGP_{NP} Dose Dependent

Protein	Dose	SUM	STDEV
**PARP**	0 µg/mL	1.00	0.00
	5 µg/mL	0.94	0.15
	10 µg/mL	0.72	0.38
	15 µg/mL	0.50	0.38
**Casp-9**	0 µg/mL	1.00	0.00
	5 µg/mL	1.11	0.14
	10 µg/mL	1.13	0.19
	15 µg/mL	1.10	0.37
**Bcl2**	0 µg/mL	1.00	0.00
	5 µg/mL	1.11	0.06
	10 µg/mL	0.82	0.09
	15 µg/mL	0.65	0.11
**Bax**	0 µg/mL	1.00	0.00
	5 µg/mL	1.43	0.23
	10 µg/mL	1.60	0.13
	15 µg/mL	1.74	0.42
